# Impacts of commingling on health and productive responses of beef heifers during feedlot receiving

**DOI:** 10.1093/tas/txaa102

**Published:** 2020-12-22

**Authors:** Jacob B Wiegand, Reinaldo F Cooke, Alice P Brandão, Kelsey M Schubach, Eduardo A Colombo, Consuelo Sowers, Glenn C Duff, Vinicius N Gouvêa

**Affiliations:** 1 Department of Animal Science, Texas A&M University, College Station, TX; 2 Clayton Livestock Research Center, New Mexico State University, Clayton, NM

## INTRODUCTION

Commingling is widely accepted as a critical stressor in feedlot systems and typically occurs shortly after major stressful events such as weaning and road transport (Cooke, 2017). When cattle from various sources are commingled in the same pen, social hierarchy is destabilized and psychological stress reactions are provoked until social structure is reestablished (Loerch and Fluharty, 1999). Hence, commingling can be perceived by cattle as an acute and/or chronic stressor depending upon how much time is required for social restabilization. Although there is a plethora of epidemiologic research recognizing commingling as a risk factor for bovine respiratory disease (BRD) in commercial feedlots (Taylor et al., 2010), few experimental studies have attempted to examine the magnitude of commingling-induced stress and its consequences to immunocompetence and productivity of receiving cattle.

Ribble et al. (1998) surveyed receiving yards that commingle cattle and reported that pens with fewer cattle sources had reduced incidence of BRD compared with pens containing cattle from a larger number of sources. These authors, however, did not quantify number of sources nor evaluated performance and immune responses to commingling. In fact, no experimental research has investigated if number of cattle sources being commingled impact resultant stress, immunocompetence, and productive responses of receiving cattle. To fulfill this lack in knowledge, we hypothesized that commingling will elicit stress responses that impact immunocompetence and performance of feedlot receiving cattle, and such outcomes intensify according to the number of cattle sources mixed within the receiving pen. Therefore, this experiment compared physiological, health, and performance responses of beef heifers assigned to different commingling schemes (1, 2, or 4 sources per pen) during a 56-d feedlot receiving period.

## MATERIALS AND METHODS

This experiment was conducted at the New Mexico State University—Clayton Livestock Research Center (Clayton, NM). All animals were cared for in accordance with acceptable practices and experimental protocols reviewed and approved by the New Mexico State University—Institutional Animal Care and Use Committee.

### Animals and Treatments

Ninety-six recently weaned Angus-influenced heifers were purchased from a commercial auction facility (Cattlemen’s Livestock Commission Company, Dalhart, TX). Heifers originated from four cow–calf ranches and were reared in the same herd within each ranch since birth. On the day of purchase (day −2; 0800 h), heifers were loaded by source into commercial livestock trailers (Legend 50’ cattle liner; Barrett LLC., Purcell, OK) at the auction yard and transported for 8 h to stimulate the stress of a long-haul (Cooke, 2017). Heifers were not mixed with cohorts from other sources prior to and at the auction yard. Heifers were unloaded at the Clayton Livestock Research Center on day −2, arrival shrunk body weight (BW) was recorded, and heifers were maintained in four paddocks by source with ad libitum access to hay, water, and a mineral supplement for 36 h.

On day 0 of the experiment, heifers were ranked by source and shrunk BW and allocated to 1 of 24 drylot pens (10 × 5 m; four heifers per pen) containing: 1) heifers from a single source (NOCOM, *n* = 8), 2) heifers from two sources (COM2, *n* = 8), or 3) heifers from four sources (COM4, *n* = 8). From days 0 to 55, heifers had free-choice access to water and RAMP (Cargill, Dalhart, TX), which was offered once daily (0800) in a manner to yield 10% residual orts. Heifers were vaccinated and administered anthelmintic on day 0 (Lopez et al., 2018).

### Sampling and Laboratorial Analyses

Heifer full BW was recorded on days 0, 6, 13, 27, 41, and 55. Shrunk BW (after 16 h of water and feed withdrawal) was also collected on day 56 for average daily gain (ADG) calculation, using shrunk BW on day −2 as initial BW. Feed intake (dry matter basis) from each pen was evaluated daily, divided by the number of heifers within each pen, and expressed as kg per heifer per day. Total BW gain and feed intake of each pen were used for feed efficiency (G:F) calculations. Heifers were observed daily for BRD signs according to the DART system (Zoetis, Florham Park, NJ) and received antimicrobial treatment as in Lopez et al. (2018). Animals were removed from the experiment if a fourth medical treatment was warranted.

Blood samples were collected from all heifers on days 0, 6, 13, 27, 41, and 55 into commercial blood collection tubes (Vacutainer, 10 mL; Becton Dickinson, Franklin Lakes, NJ) containing freeze-dried sodium heparin for plasma collection. All blood samples were placed immediately on ice, centrifuged (2,500 × *g* for 30 min; 4 °C) for plasma harvest and stored at −80 °C on the same day of collection. Plasma samples were analyzed for haptoglobin and cortisol as described by Colombo et al. (2019), with intra and interassay CVs ≤ 7%.

### Statistical Analysis

Pen was considered the experimental unit for all analyses. Quantitative data were analyzed using the MIXED procedure of SAS (SAS Inst. Inc., Cary, NC), whereas binary data were analyzed using the GLIMMIX procedure of SAS (SAS Inst. Inc.). All data were analyzed using Satterthwaite approximation to determine the denominator df for tests of fixed effects, with pen(treatment) and heifer(pen) as random variables but for feed intake and G:F that used pen(treatment) as the random variable. Model statements contained the effects of treatment, day, and the treatment × day interaction for repeated measures, in addition to source as independent variable. Plasma variables were also analyzed using results from day 0 as independent covariate. The specified term for all repeated statements was day, with pen(treatment) as subject for feed intake and heifer(pen) as subject for all other analyses. The covariance structure used was first-order autoregressive, which provided the smallest Akaike information criterion. Results are reported as least square means, or covariately adjusted least square means for plasma variables, and separated using PDIFF. Significance was set at *P* ≤ 0.05 and tendencies at *P* > 0.05 and ≤ 0.10. Results are reported according to main effects if no interactions were significant, or according to the highest order interaction detected.

## RESULTS

**Figure 1.**
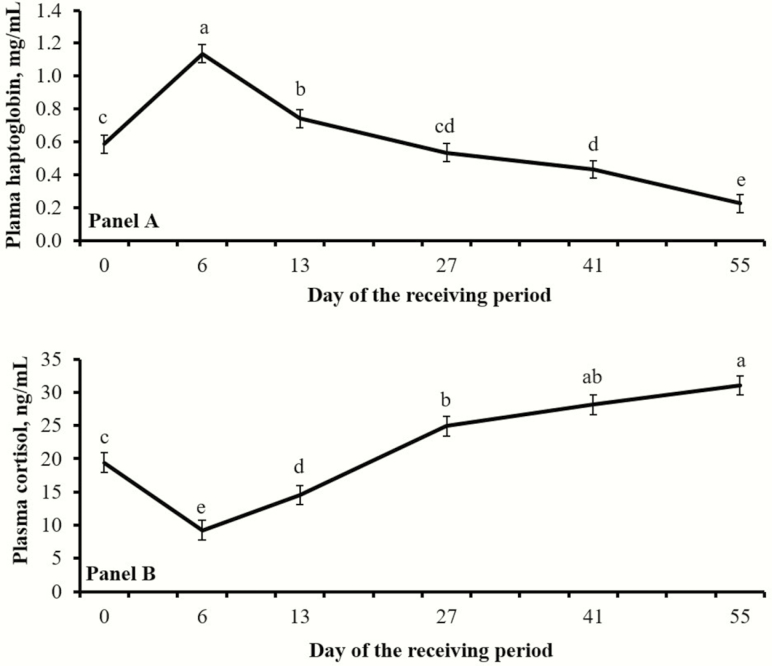
Concentrations of plasma haptoglobin (A) and cortisol (B) from beef heifers relative to feedlot arrival (day 0). Day effects were detected (*P* < 0.01) for both variables. Days with different superscripts (a,b,c,d,e) differ (*P* ≤ 0.05).

As designed, initial shrunk BW (day 0) was similar (*P* = 0.99) between treatments (Table 1). Average daily gain and final shrunk BW did not differ (*P* ≥ 0.83) between treatments (Table 1). No treatment effects were detected (*P* ≥ 0.77) for feed intake and G:F (Table 1), as well as plasma concentrations of haptoglobin and cortisol (Table 2). Day effects were detected (*P* < 0.01) for both plasma variables (Figure 1).

**Table 1.** Performance parameters of beef heifers commingled (COM2 = 2 sources; *n* = 8; COM4 = 4 sources, *n* = 8) or not (NOCOM = single source, *n* = 8) with cohorts from different cow–calf sources during a 56-d feedlot receiving^1^

Item	NOCOM	COM2	COM4	SEM	*P*-value
Initial body weight, kg	240	239	240	7	0.99
Final body weight, kg	287	290	286	7	0.93
Average daily gain, kg/d	0.855	0.886	0.818	0.077	0.80
Feed intake, kg/d	6.48	6.55	6.32	0.23	0.77
Feed efficiency, g/kg	140	143	135	10	0.82

^1^Heifer shrunk body weight was recorded on day −2 (initial; upon arrival) and day 56 (final; after 16 h of water and feed withdrawal). Feed intake was recorded daily from days 0 to 55 by measuring offer and refusals from each pen, divided by the number of heifers within each pen, and expressed as kg per heifer/d. Feed efficiency was calculated using total feed intake from days 0 to 55, and body weight gain of each pen from days −2 to 55.

**Table 2.** Health and physiological responses from beef heifers commingled (COM2 = 2 sources; *n* = 8; COM4 = 4 sources, *n* = 8) or not (NOCOM = single source, *n* = 8) with cohorts from different cow-calf sources during a 56-d feedlot receiving^1^

Item	NOCOM	COM2	COM4	SEM	*P*-value
Incidence of BRD signs, %	53.1	68.7	56.2	9.7	0.49
Sick cattle requiring 2 treatments	26.2	28.8	29.6	10.7	0.97
Sick cattle requiring ≥3 treatments	0.00	11.9	20.2	7.50	0.17
Number of antimicrobial treatments required	1.22	1.49	1.54	0.18	0.45
Mortality, %	9.37	0.00	3.12	3.54	0.17
Physiological variables					
Plasma cortisol, ng/mL	22.0	21.1	21.3	1.7	0.93
Plasma haptoglobin, mg/mL	0.625	0.619	0.656	0.067	0.91

^1^Heifers were observed daily for BRD signs according to the DART system (Zoetis, Florham Park, NJ), and received antimicrobial treatment as in Lopez et al. (2018). Blood samples were collected on days 0, 6, 13, 27, 41, and 55. Values obtained on day 0 were used as covariate within each respective analysis.

**Figure 2.**
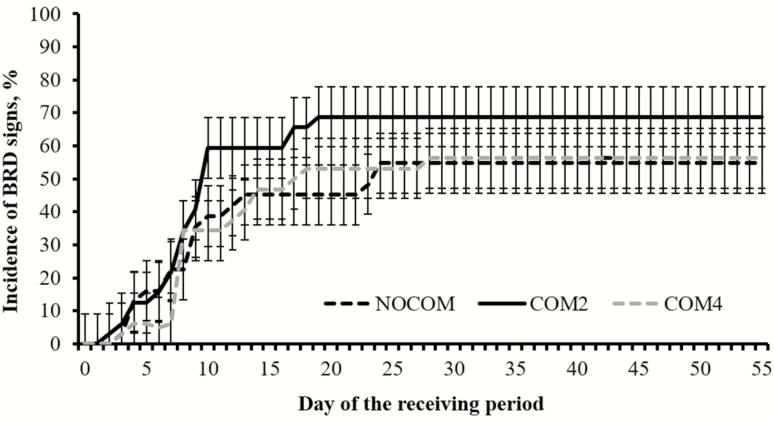
Cumulative incidence of bovine respiratory disease (BRD) symptoms beef heifers commingled (COM2 = 2 sources; *n* = 8; COM4 = 4 sources, *n* = 8) or not (NOCOM = single source, *n* = 8) with cohorts from different cow–calf sources during a 56-d feedlot receiving. Heifers were observed daily for BRD signs according to the DART system (Zoetis, Florham Park, NJ) and received antimicrobial treatment as in Lopez et al. (2018). No treatment effects were noted (*P* ≥ 0.46).

No treatment differences were noted (*P* ≥ 0.49) for morbidity and mortality during the experiment (Table 2; Figure 2). A similar (*P ≥* 0.97) proportion of NOCOM, COM2, and COM4 heifers diagnosed with BRD required at least two treatments to recover from sickness. In turn, a third treatment was not required for NOCOM heifers, which was less compared with COM4 (*P* = 0.05) and similar (*P* = 0.23) compared with COM2 heifers (Table 2).

## DISCUSSION

Heifers used in this experiment were considered high risk as their management and health history were not fully known (Wilson et al., 2017). Heifers were exposed to the stress of weaning, transportation, commingling, vaccination, and feedlot entry within a 72-h period, which are known to impact cattle immunocompetence and performance (Cooke, 2017). The daily changes noted for plasma cortisol and haptoglobin (Figure 2) support that heifers experienced an adrenocortical and subsequent acute phase protein response elicited by transportation and feedlot entry (Cooke, 2017). Stress-induced inflammation increases the susceptibility of receiving cattle to BRD (Cooke, 2017), corroborating the substantial incidence of BRD observed herein (Table 2; Figure 1).

We speculated that commingling would further increase the cortisol and haptoglobin responses during feedlot receiving, reducing performance and increasing BRD as sources of cattle commingled increased. However, no differences in ADG, feed intake, G:F, BRD incidence, and plasma concentrations of cortisol and haptoglobin were noted among treatments during the 56-d receiving period. The only health benefit observed by not commingling was that NOCOM heifers diagnosed with BRD did not require a third treatment to recover from sickness, whereas ≥11% of commingled heifers required such treatment. Collectively, this experiment failed to observe major negative impacts of commingling high-risk heifers on their performance and health responses during a 56-d receiving period.

The current knowledge associating commingling and BRD incidence in receiving cattle is mostly based on epidemiological research (Taylor et al., 2010) or results confounded with cattle origin (Arthington et al., 2003; Step et al., 2008). To our understanding, this is the first experimental research balanced for cattle source and different levels of commingling, and investigating productive, physiological, and health consequences. To achieve such design, four cattle sources were chosen, and four heifers per pen were utilized, so treatments and experimental units were balanced for heifer source. Moreover, either one or two heifers from the same source (COM4 and COM2, respectively) were housed together in commingled pens. Cattle are social animals and may form group sizes containing 20 to 100 individuals (Bouissou et al., 2001), whereas epidemiological studies reporting increased BRD in commingled cattle surveyed feedlots with ≥50 animals per pen. Hence, lack of treatment differences noted herein may be associated with the number of heifers assigned to each pen, which limited the disruption of pre-existing social groups and hierarchies within comingled pens.

## IMPLICATIONS

This experimental model fully represented the stress and health challenges experienced by commercial cattle during feedlot receiving, resulting in substantial BRD incidence and morbidity. However, commingling heifers from different cow–calf sources did not affect performance, physiological, and health responses as hypothesized. Perhaps the number of heifers assigned to commingled pens, and resultant pre-existing social groups, was not sufficient stimulate the stress reactions by social destabilization. Therefore, experimental research investigating commingling high-risk cattle during feedlot receiving are still warranted, particularly designs using pens sizes representative of commercial feedyards.
